# Beliefs and perceptions of electronic cigarettes among medical staff in respiratory departments of Fujian Province, China, in 2021

**DOI:** 10.18332/tid/156038

**Published:** 2022-12-12

**Authors:** Pengxiang Huang, Wanyu Zheng, Yangzhen Shi, Liyong Shi, Weijing Wu, Xiaoping Lin, Xiaohong Zeng, Fei He, Maolin Liu, Xiaoyang Chen

**Affiliations:** 1 Department of Pulmonary and Critical Care Medicine, The Second Affiliated Hospital of Fujian Medical University, Quanzhou, China; 2 Department of Pulmonary and Critical Care Medicine, Jinjiang Municipal Hospital, Quanzhou, China; 3 Department of Epidemiology and Health Statistics, School of Public Health, Fujian Medical University, Fuzhou, China

**Keywords:** electronic cigarettes, medical staff, respiratory department, smoking cessation

## Abstract

**INTRODUCTION:**

Due to the popularity of e-cigarettes, more and more patients ask about e-cigarettes, and it is particularly important to understand doctors’ beliefs and perceptions on e-cigarettes. The aim was to evaluate the belief and perception of electronic cigarettes among medical staff in the respiratory department of medical institutions located in Fujian Province.

**METHODS:**

The electronic questionnaires were conveyed to the medical staff of the respiratory department in Fujian Province during March to April 2021. Descriptive statistics were calculated for all questions, and the relationship between relevant factors and the perception of e-cigarette-related statements was analyzed by logistic regression analysis.

**RESULTS:**

Among 1028 medical staff in the respiratory departments of Fujian Province, 90.5% of medical staff agreed that electronic cigarettes are harmful to the human body; 61.4% of medical staff agreed that e-cigarettes cannot be regarded as a type of smoking cessation treatment; 71.7% of medical staff agreed that e-cigarettes could be a ‘gateway’ to other tobacco use; and 69.2% of medical staff agreed that electronic cigarettes are in ‘Three No’ states. The multivariate logistic regression analysis showed that the respondents’ perception of ‘e-cigarettes cannot be regarded as a type of smoking cessation treatment’ were related to gender, professional title and whether they participated in the cessation clinic.

**CONCLUSIONS:**

The medical staff of the respiratory department in Fujian Province put more emphasis on the adverse effects of e-cigarettes on health, but lack the cognition of the effect of e-cigarette smoking cessation. In order to better carry out smoking cessation work, it is necessary to strengthen the training of respiratory medical staff at all levels of medical institutions on e-cigarette knowledge.

## INTRODUCTION

China is the world’s largest producer and consumer of tobacco, with more than 300 million smokers and 740 million people affected by secondhand smoke^1^. The prevalence of tobacco use in China is high, which is a significant burden to society. However, in addition to traditional tobacco, e-cigarettes have gradually become a serious public health problem^2^. An e-cigarette is a device that produces and transports inhaled aerosols containing nicotine. The original intention of the invention of e-cigarettes was to help quit smoking as a less harmful alternative to tobacco. However, e-cigarettes have become popular among young people because of their great attraction in product design, taste, marketing and security^3,4^.

Studies have shown that e-cigarette aerosols contain nicotine, carbonyl compounds, nitrosamines, lead, cadmium and other harmful substances^5,6^, but the content of these harmful substances is significantly lower than that of tobacco^7^. The industry has adopted active marketing strategies on media and social networks to promote e-cigarettes as a healthier alternative to cigarettes^8,9^. However, harm reduction does not mean ‘no risk’. The outbreak of e-cigarette or vaping use-associated lung injury (EVALI) in the United States in 2019 raised public concern about the security of e-cigarettes^10^, although closely related to vitamin E additives^11^. In addition, more and more evidence^6,12-14^ shows that e-cigarettes can damage the respiratory system, cardiovascular system, DNA, etc. Especially for adolescents, e-cigarettes have adverse effects on brain development and cognition^15,16^. E-cigarette product regulation is not consistently enforced; in some cases, the labels do not match the products^17^.

In addition, the role of e-cigarettes in smoking cessation is still a controversial issue. A clinical trial published in 2019 found that using e-cigarettes to quit smoking is more effective than nicotine substitutes^18^. Walker et al.^19^ found that the use of nicotine patches combined with e-cigarettes can increase the rate of smoking cessation. However, some studies have come to the opposite conclusion, they believe that the use of e-cigarettes not only cannot help quit smoking but there is a dual use of traditional cigarettes and e-cigarettes, and the use of e-cigarettes quit smoking rate will be lower^20,21^. Ruifeng et al.^22^ found in the 2017–2019 PATH study cohort that using e-cigarettes did not improve the success rate of smoking cessation or prevent recurrence. The above studies on e-cigarettes mostly have problems such as short follow-up time and the number of studies is small. Due to the harmfulness of long-term use and the uncertainty of smoking cessation efficacy, e-cigarettes are not currently used as a routine cessation method^23,24^.

Since 2014, e-cigarettes have become the most commonly used tobacco product among US teens^25^. The survey results released by the Chinese Center for Disease Control and Prevention show that 2.7% of junior high school students and 3% of high school students in China have used e-cigarettes^26^. Studies have shown that the use of nicotine-containing e-cigarettes can make adolescents addicted to nicotine and later more likely to gradually transition to traditional tobacco to meet the demand for nicotine, known as the ‘gateway effect’^27^. In the face of the rapid development of e-cigarettes and the severe prevalence of youth e-cigarette use, e-cigarette regulation has lagged behind^24^. The WHO reported that 84 countries do not regulate ENDS at all, while of the 79 countries that regulate the products, only nine have banned or restricted flavors^28^. So far, China’s national supervision of e-cigarettes is mainly to ban e-cigarette sales to minors and stop the selling and advertising of e-cigarettes through online channels^29^.

Due to the popularity of e-cigarettes, more and more patients are asking about e-cigarettes, and it is essential to understand doctors’ beliefs and perceptions of e-cigarettes^30^. Andrew et al.^31^ reported that among 204 pulmonary physicians surveyed in the United States in 2016, 65% agreed that e-cigarettes had adverse effects on health. With the continuous reports of e-cigarette adverse events and the progress of e-cigarette-related research in recent years^32^, the medical staff’s understanding of e-cigarettes has gradually changed. The theoretical knowledge and practical skills of respiratory care staff affect the progression of tobacco control. To understand the belief and perception of e-cigarettes among respiratory medical staff in Fujian Province, we surveyed respiratory medical staff in 89 medical institutions in Fujian Province from March to April 2021.

## METHODS

### Research objective

This study aimed to investigate the belief and perceptions on e-cigarettes of respiratory medical staff in Fujian Province. The Ethical Evaluation Committee approved the study of the 2nd Affiliated Hospital of Fujian Medical University in China (ethical code B2020-467). Inclusion criteria were: 1) on-the-job respiratory doctors and nurses; and 2) the ability to understand and voluntarily participate in the questionnaire survey. Exclusion criteria were: people who do not know about e-cigarettes. A total of 1028 respiratory medical staff (doctors and nurses) from 89 provincial, municipal, and county hospitals in Fujian Province, China, participated in the study. There were 276 males (26.8%) and 752 females (73.2%); there were 448 respiratory physicians (43.6%) and 580 respiratory nurses (56.4%).

### Investigation method

By contacting the directors of respiratory departments of 89 provincial, municipal and county hospitals in Fujian Province in advance, they were informed of the purpose and content of the survey, and their understanding and cooperation were obtained. From March to April 2021, these respiratory directors distributed the digital questionnaire to medical staff who met the inclusion criteria through the Questionnaire Star (online platform www.wjx.cn). Participants completed the questionnaire independently during office hours, and the respiratory director needed to avoid the filling out of the questionnaires. This is an anonymous survey, and the results are confidential. Finally, 1028 medical staff participated in the online questionnaire survey, and the questionnaires were valid.

### Investigation content

The questionnaire was compiled based on the literature related to e-cigarettes^31-33^. In addition to demographic information, to assess the respondents’ perception of e-cigarettes, the respondents were asked whether they agreed with the following statements: ‘e-cigarettes are harmful to the human body’; ‘ e-cigarettes cannot be regarded as a type of smoking cessation treatment’; ‘e-cigarettes could be a 'gateway' to other tobacco use’; and ‘e-cigarettes are in 'Three No' states’, using the response categories of ‘agree’, ‘disagree’ or ‘unclear’.

### Index definitions

*E-cigarettes cannot be regarded as a type of smoking cessation treatment*

It is believed that electronic cigarettes cannot replace traditional tobacco to help quit smoking.

*E-cigarettes could be a ‘gateway’ to other tobacco use*

It is believed that e-cigarettes may make young people who do not smoke eventually become consumers of traditional cigarettes.

*E-cigarettes are in ‘Three No’ states*

China’s e-cigarettes are in the ‘Three No’ states. It means that there is no product standard, no quality supervision, and no safety evaluation.

### Statistical analysis

After the questionnaire was exported from the network, SPSS 23.0 software was used for statistical analysis. The number, percentage and rate of use cases are statistically described. Univariate logistic regression analysis was used to explore the relationship between the level of perception about electronic cigarette-related statements and demographic characteristics. Multivariate logistic regression analysis was used to explore the relationship between the level of perception of ‘e-cigarettes cannot be regarded as a type of smoking cessation treatment’ and demographic characteristics.

Gender, age, education level, medical staff type, hospital level title, professional titles and whether to participate in cessation clinic, were included in the multivariate logistic regression analysis model. The forward LR method was used to construct the multivariate logistic regression analysis model. The significance was determined as p<0.05 (two-tailed), and the confidence limit was 95%.

## RESULTS

### Basic information

A total of 1028 respiratory medical staff (doctors and nurses) from 89 provincial, municipal, and county hospitals in Fujian Province, China, participated in the study and the questionnaires were valid. There were 448 respiratory physicians, accounting for 43.6%, and 580 respiratory nurses, accounting for 56.4%. Most participants were in the age group of 20–49 years, accounting for 95.1% of all responses. The professional titles were mainly junior and intermediate (83.5%). The education level is primarily undergraduate and junior college or lower (85.2%). Only 20 participants smoked (1.96%) (Table 1).

**Table 1:** Basic information of respondents in the respiratory department in Fujian Province, 2021 (N=1028)

*Characteristics*	*n*	*%*
**Gender**		
Male	276	26.8
Female	752	73.2
**Age** (years)		
20–34	672	65.4
35–49	306	29.8
50–59	49	4.8
≥60	1	0.1
**Education level**		
Junior college or lower	405	39.4
Undergraduate	471	45.8
Postgraduate or higher	131	12.7
**Medical staff type**		
Doctor	21	2
Physician	448	43.6
Nurse	580	56.4
**Professional titles**		
Junior	590	57.4
Intermediate	268	26.1
Senior	170	16.5
**Hospital level**		
Tertiary grade A	565	55
Tertiary grade B or C	159	15.5
Secondary grade	277	26.9
Private	19	1.8
Other	8	0.8
**Smoking status**		
Non-smoker	993	96.6
Former smoker	19	1.8
Current smoker	16	1.6
**Exposure to secondhand smoke**		
Often	174	16.9
Sometimes	762	74.1
Never	92	8.9
**Smoking cessation clinic**		
Yes	379	36.9
No	649	63.1
**Total**	1028	100

### Beliefs and perceptions of electronic cigarettes

Among 1028 medical staff in the respiratory department in Fujian Province, 90.5% of medical staff agreed that e-cigarettes are harmful to the human body; 61.4% of medical staff agreed that e-cigarettes could not be regarded as a smoking-cessation treatment; 71.7% of medical staff agreed that e-cigarettes could be a ‘gateway’ to other tobacco use; and 69.2% of medical staff agreed that electronic cigarettes are in ‘Three No’ states (Table 2).

**Table 2:** Belief and perception of electronic cigarettes among medical staff in the respiratory department in Fujian Province, 2021 (N=1028)

*Agreed with the following statements*	*n (%)*
E-cigarettes are harmful to the human body	930 (90.5)
E-cigarettes cannot be regarded as a type of smoking cessation treatment^a^	631 (61.4)
E-cigarettes could be a ‘gateway’ to other tobacco use^b^	737 (71.7)
E-cigarettes are in ‘Three No’ states^c^	711 (69.2)

a It is believed that electronic cigarettes cannot replace traditional tobacco to help quit smoking.

b It is believed that e-cigarettes may make young people who do not smoke eventually become consumers of traditional cigarettes.

c It means that there is no product standard, no quality supervision, and no safety evaluation.

### Perception of ‘e-cigarettes are harmful to the human body’

Regarding agreement rates of ‘e-cigarettes are harmful to the human body’, there were differences among different gender, age, education level, medical staff type, and professional titles. The agreement rates of males aged 35–49 years, postgraduate or higher, physician, and senior professional title, were higher than those of females aged 20–34 years, junior college or lower, nurse, and junior professional title. There was no difference in the agreement rates of ‘e-cigarettes are harmful to the human body’ at different hospital levels (Table 3).

**Table 3:** Univariate logistic regression analysis of factors affecting the perception of ‘e-cigarettes are harmful to the human body’ among medical staff in the respiratory department in Fujian Province, 2021 (N=1028)

*Characteristics*	*E-cigarettes are harmful to the human body (‘agree’) n (%)*	*OR (95% CI)*	*p*
**Gender**			
Female (Ref.)	670 (89.1)	1	
Male	260 (94.2)	1.99 (1.14–3.46)	0.015
**Age** (years)			
20–34 (Ref.)	596 (88.7)	1	
35–49	288 (94.1)	2.04 (1.20–3.48)	0.009
≥50	46 (92.0)	1.47 (0.51–4.19)	0.475
**Education level**			
Junior college or lower (Ref.)	356 (87.9)	1	
Undergraduate	430 (91.3)	1.44 (0.93–2.24)	0.100
Postgraduate or higher	144 (94.7)	2.48 (1.15–5.36)	0.021
**Medical staff type**			
Nurse (Ref.)	510 (87.9)	1	
Physician	420(93.8)	2.06 (1.30–3.25)	0.002
**Hospital level**			
Tertiary grade A (Ref.)	505 (89.4)	1	
Tertiary grade B or C	144 (90.6)	1.14 (0.63–2.07)	0.665
Secondary grade/private/other	281 (92.4)	1.45 (0.88–2.40)	0.146
**Cessation clinic**			
No (Ref.)	580 (89.4)	1	
Yes	350 (92.3)	1.44 (0.91–2.26)	0.118
**Professional titles**			
Junior (Ref.)	522 (88.5)	1	
Intermediate	246 (91.8)	1.46 (0.88–2.41)	0.144
Senior	162 (95.3)	2.64 (1.24–5.60)	0.012

### Perception of ‘e-cigarettes cannot be regarded as a type of smoking cessation treatment’

Regarding the agreement rates of ‘e-cigarettes cannot be regarded as a type of smoking cessation treatment’, there were differences among different gender, age, education level, medical staff type, professional titles, and whether to participate in a cessation clinic. The agreement rates of males aged 35–49 years and >50 years, undergraduate and postgraduate or higher, physician, intermediate and senior professional titles and participating in cessation clinic, were higher than those of females aged 20–34 years, junior college or lower, nurse, junior professional title and not participating in cessation clinic. There was no difference in the agreement rates of ‘e-cigarettes cannot be regarded as a type of smoking cessation treatment’ at different hospital levels (Table 4).

**Table 4:** Univariate logistic regression analysis of factors affecting the perception of ‘e-cigarettes cannot be regarded as a type of smoking cessation treatment’ among medical staff in the respiratory department in Fujian Province, 2021 (N=1028)

*Characteristics*	E-cigarettes cannot be regarded as a type of smoking cessation treatment* (‘agree’) n (%)	*OR (95% CI)*	*p*
**Gender**			
Female (Ref.)	414 (55.1)	1	
Male	217 (78.6)	3.00 (2.18–4.14)	<0.001
**Age** (years)			
20–34 (Ref.)	371 (55.2)	1	
35–49	221 (72.2)	2.11 (1.57–2.83)	<0.001
≥50	39 (78.0)	2.88 (1.45–5.71)	0.003
**Education level**			
Junior college or lower (Ref.)	207 (51.1)	1	
Undergraduate	313 (66.5)	1.90 (1.44–2.49)	<0.001
Postgraduate or higher	111 (73.0)	2.59 (1.72–3.89)	<0.001
**Medical staff type**			
Nurse (Ref.)	288 (49.7)	1	
Physician	343 (76.6)	3.31 (2.52–4.35)	<0.001
**Hospital level**			
Tertiary grade A (Ref.)	347 (61.4)	1	
Tertiary grade B or C	98 (61.6)	1.01 (0.70–1.45)	0.960
Secondary grade/private/other	186 (61.2)	0.99 (0.74–1.32)	0.947
**Cessation clinic**			
No (Ref.)	366 (56.4)	1	
Yes	265 (69.9)	1.8 (1.37–2.35)	<0.001
**Professional titles**			
Junior (Ref.)	312 (52.9)	1	
Intermediate	183 (68.3)	1.92 (1.42–2.60)	<0.001
Senior	136 (80.0)	3.56 (2.37–5.37)	<0.001

* It is believed that electronic cigarettes cannot replace traditional tobacco to help quit smoking.

Gender, age, education level, medical staff type, hospital level title, professional titles and whether to participate in cessation clinic, were included in the multivariate logistic regression analysis model. The forward LR method was used to construct the multivariate logistic regression analysis model. In terms of the agreement rates of ‘e-cigarettes cannot be regarded as a type of smoking cessation treatment’, multivariate logistic regression analysis showed that men’s agreement rate (AOR=2.12; 95% CI: 1.49–3.02) was higher than that of women. The agreement rate of medical staff with intermediate titles (AOR=1.56; 95% CI: 1.14–2.15) and medical staff with senior titles (AOR=2.20; 95% CI: 1.40–3.47) was higher than that of medical staff with junior titles. The agreement rate of medical staff participating in the smoking cessation clinic (AOR=1.33; 95% CI: 1.00–1.78) was higher than those who did not participate in the smoking cessation clinic (Table 5).

**Table 5:** Multivariate logistic regression analysis^a^ of factors affecting the perception of ‘e-cigarettes cannot be regarded as a type of smoking cessation treatment’* among medical staff in the respiratory department in Fujian Province, 2021 (N=1028)

*Characteristics*	*β*	*SE*	*Wald χ^2^*	*p*	*AOR (95% CI)*
**Gender**					
Female (Ref.)					1
Male	0.753	0.180	17.586	<0.001	2.12 (1.49–3.02)
**Cessation clinic**					
No (Ref.)					1
Yes	0.288	0.146	3.869	0.049	1.33 (1.00–1.78)
**Professional titles**					
Junior (Ref.)					1
Intermediate	0.447	0.162	7.660	0.006	1.56 (1.14–2.15)
Senior	0.790	0.231	11.714	0.001	2.20 (1.40–3.47)

AOR: adjusted odds ratio; adjusted for gender, age, education level, medical staff type, hospital level title, professional titles, and whether to participate in cessation clinic.

* It is believed that electronic cigarettes cannot replace traditional tobacco to help quit smoking.

a The forward LR method was used to construct the multivariate logistic regression analysis model.

### Perception of ‘e-cigarettes could be a 'gateway' to other tobacco use’

In terms of the agreement rates of ‘e-cigarettes could be a 'gateway' to other tobacco use’, there were differences among different gender, age, education level, medical staff type, professional titles, and whether to participate in cessation clinics. The agreement rates of males aged 35–49 years, postgraduate or higher, physician, intermediate and senior professional titles, and participating in cessation clinic, were higher than those of females aged 20–34 years, junior college or lower, nurse, junior professional title and not participating in cessation clinic. There was no difference in the agreement rates of ‘e-cigarettes could be a 'gateway' to other tobacco use’ at different hospital levels (Supplementary file Table 1).

### Perception of ‘e-cigarettes are in 'Three No' states’

In terms of the agreement rates of ‘e-cigarettes are in 'Three No' states’, there were differences among different gender, age, education level, medical staff type, professional titles, and whether to participate in cessation clinics. The agreement rates of males aged 35–49 years, undergraduate and postgraduate or higher, physician, intermediate and senior professional titles, and participating in cessation clinic, were higher than those of females aged 20–34 years, junior college or lower, nurse, junior professional title, and not participating in cessation clinic. There was no difference in the agreement rates of ‘e-cigarettes are in 'Three No' states’ at different hospital levels (Supplementary file Table 2).

## DISCUSSION

Through a questionnaire survey of 1028 respiratory medical staff in 89 hospitals in Fujian Province, we found that 90.5% of the respondents agreed that e-cigarettes were harmful to the human body, which was similar to the survey results of Korean lung cancer specialists^33^. Yitian et al.^34^ reported that 46.3% of 1023 Chinese doctors in 2018 agreed that e-cigarettes were harmful to health. Previous studies showed that 65% of 204 US pulmonary physicians agreed that e-cigarettes had adverse effects on health^31^. The results of this survey indicate that the medical staff of the respiratory department in Fujian Province have a high perception of the harm of e-cigarettes, which may be related to the adverse events caused by e-cigarettes, including the ‘American outbreak of EVALI’ reported by the media in recent years^10^. Medical staff in the respiratory department may also be more concerned about the health problems caused by e-cigarettes than other specialists. Research shows that 11.7% of Chinese e-cigarette users use them because they believe that e-cigarettes are less harmful than cigarettes^29^. Medical staff should increase public awareness of the harms of e-cigarettes, which could reduce the use of e-cigarettes. The World Health Organization pointed out that ‘e-cigarettes are harmful and must be managed’^35^. A series of strategies like the enactment of e-cigarettes-related laws, strengthening the supervision of e-cigarettes, and establishing a reporting and monitoring system for adverse events of e-cigarettes could significantly reduce the harm caused by e-cigarettes.

The survey found that only 61.4% of the medical staff in the respiratory department of Fujian Province believed that e-cigarettes could not be regarded as a type of smoking cessation treatment. In Korea, Dong et al.^33^ found that only 21.6% of Korean lung cancer specialists agreed that e-cigarettes could help quit smoking. Conversely, research shows that 70.4% of pulmonary physicians in the US agreed that e-cigarettes could help quit smoking^31^. These surveys show great differences in medical staff’s views on using e-cigarettes to quit smoking. Cochrane’s review of published literature found moderate-certainty evidence that e-cigarettes with nicotine increase quit rates compared to nicotine replacement therapy^36^. Dong et al.^33^ found that most Korean lung cancer specialists understanding of e-cigarettes mainly comes from the media, rather than professional, scientific resources. This survey found that medical staff with higher professional titles or participating in smoking cessation clinics were more likely to think that e-cigarettes could not help to stop quit smoking. This may also be because medical staff in the respiratory department of Fujian Province did not pay attention to relevant scientific research on e-cigarettes, resulting in a deviation in understanding the effect of e-cigarettes on smoking cessation. The Tobacco Control Office of the Chinese Center for Disease Control and Prevention survey found that 46.2% of e-cigarette users used e-cigarettes to quit smoking. However, the level of dual use among e-cigarette users is high, and the quit rate is low, which means that e-cigarettes have no cessation effect at the population level in China^29^. Similarly, Flacco et al.^24^ found in a six-year (2013–2019) observational study that using e-cigarettes did not improve cessation^21^. Most countries do not use e-cigarettes as a routine smoking cessation method. The safety and effectiveness of e-cigarettes as a smoking cessation therapy must be based on high-quality randomized controlled studies. Respiratory medical staff in China should pay more attention to the progress of scientific research related to e-cigarettes. Still, at present, they should be cautious about patient consultations on using e-cigarettes to quit smoking.

The ‘gateway effect’ of e-cigarettes also deserves our attention^37^. The survey found that 71.7% of respiratory medical staff in Fujian Province agreed that e-cigarettes could be a ‘gateway’ to other tobacco use. In contrast, only 41.2% of 1023 Chinese doctors in 2018 decided e-cigarettes could be a ‘gateway’ to other tobacco use^34^. In addition to the ‘gateways’ effect, some studies have found that teens who use e-cigarettes are more likely to have behaviors such as alcohol abuse, drug abuse, and sexual behaviour^38^. Medical staff should pay particular attention to the popular trend of e-cigarettes among teenagers. They should educate teenagers about e-cigarettes through various channels such as campus, community and network media because reducing the use of e-cigarettes among teenagers will help reduce tobacco use and the occurrence of illegal behaviors. Protecting teenagers from nicotine dependence and solving the prevalence of e-cigarettes among teenagers are essential goals of tobacco control in the future^39^.

### Strengths and limitations

To our knowledge, this study is the first to describe the belief and perception of e-cigarettes among Chinese respiratory medical staff, especially in smoking cessation clinics. But this survey has some limitations. In the design of the problem, only the medical staff’s view on electronic cigarettes was considered. Still, it was not considered whether the medical staff asked the patients about the use of electronic cigarettes and whether they provided suggestions on the electronic cigarette during the clinical diagnosis and treatment. Samples for this survey were limited to Fujian Province, so our findings cannot be extended to the broader Chinese population of respiratory medicine practitioners. The results may overestimate the cognition of e-cigarettes because the study subjects excluded medical personnel who had not heard of e-cigarettes.

## CONCLUSIONS

The medical staff of the respiratory department in Fujian Province put more emphasis on the adverse effects of e-cigarettes on health but lacked cognition of the impact of e-cigarette smoking cessation. To better carry out smoking cessation work, it is necessary to strengthen the training of respiratory medical staff at all levels of medical institutions on e-cigarette knowledge. Respiratory medical staff should also pay attention to the progress of e-cigarette-related research and provide more evidence-based recommendations for patients with smoking cessation needs.

## Data Availability

The data supporting this research are available from the authors on reasonable request.
