# Supplementary material for: Beliefs and perceptions of electronic cigarettes among medical staff in respiratory departments of Fujian Province, China, in 2021
Source: Tob Induc Dis. 2022 Dec 12;20:111. doi: 10.18332/tid/156038 (PMC9743794; doi:10.18332/tid/156038)
Supplement: Supplementary file 1 [file TID-20-111-s1.pdf]

Supplementary table 1. Univariate logistic regression analysis of factors affecting the perception of " e-cigarettes could be a 'gateway' to other tobacco use " among medical staff in respiratory department in Fujian Province in 2021（N=1028）

Characteristics	E-cigarettes could be a 'gateway' to other tobacco use . ("agree") No. (%)	OR（95%CI）	*P*
**Gender**			
**Female**	520 (69.1)	1	
**Male**	217 (78.6)	1.64 (1.18-2.28)	0.003
**Age**			
**20-34**	461 (68.6)	1	
**35-49**	239 (78.1)	1.63 (1.19-2.24)	0.002
**≥50**	37 (74.0)	1.30 (0.68-2.50)	0.427
**Education**			
**Junior college or below**	276 (68.1)	1	
**Undergraduate**	340 (72.2)	1.21 (0.91-1.62)	0.192
**Postgraduate or above**	121 (79.6)	1.82 (1.17-2.85)	0.008
**Medical staff type**			
**Nurse**	396 (68.3)	1	
**Physician**	341 (76.1)	1.48 (1.12-1.96)	0.006
**Hospital level**			
**Tertiary grade A hospital**	407 (72.0)	1	
**Tertiary grade B or C hospital**	112 (70.4)	0.93 (0.63-1.36)	0.693
**Secondary grade/Private/ Other hospital**	218 (71.7)	0.98 (0.72-1.34)	0.919
**Cessation clinic**			
**No**	450 (69.3)	1	
**Yes**	287 (75.7)	1.38 (1.03-1.84)	0.029
**Professional titles**			
**Junior**	398 (67.5)	1	
**Intermediate**	199 (74.3)	1.39 (1.01-1.92)	0.045
**Senior**	140 (82.4)	2.25 (1.46-3.46)	＜0.001

Index definition: **E-cigarettes could be a 'gateway' to other tobacco use** It is believed that e-cigarettes may make young people who do not smoke eventually become consumers of traditional cigarettes.

Supplementary table 2. Univariate logistic regression analysis of factors affecting the perception of " e-cigarettes are in 'Three No' states " among medical staff in respiratory department in Fujian Province in 2021（N=1028）

Characteristics	E-cigarettes are in 'Three No' states. ("agree") No. (%)	OR（95%CI）	*P*
**Gender**			
**Female**	479 (63.7)	1	
**Male**	232 (84.1)	3.01 (2.11-4.29)	＜0.001
**Age**			
**20-34**	434 (64.6)	1	
**35-49**	238 (77.8)	1.92 (1.40-2.62)	＜0.001
**≥50**	39 (78.0)	1.94 (0.98-3.87)	0.058
**Education**			
**Junior college or below**	237 (58.5)	1	
**Undergraduate**	350 (74.3)	2.05 (1.54-2.73)	＜0.001
**Postgraduate or above**	124 (81.6)	3.14 (1.99-4.95)	＜0.001
**Medical staff type**			
**Nurse**	352 (60.7)	1	
**Physician**	359 (80.1)	2.61 (1.96-3.48)	＜0.001
**Hospital level**			
**Tertiary grade A hospital**	387 (68.5)	1	
**Tertiary grade B or C hospital**	110 (69.2)	1.03 (0.71-1.51)	0.869
**Secondary grade/Private/ Other hospital**	214 (70.4)	1.09 (0.81-1.48)	0.563
**Cessation clinic**			
**No**	421 (64.9)	1	
**Yes**	290 (76.5)	1.77 (1.32-2.35)	＜0.001
**Professional titles**			
**Junior**	370 (62.7)	1	
**Intermediate**	203 (75.7)	1.86 (1.34-2.57)	＜0.001
**Senior**	138 (81.2)	2.56 (1.69-3.90)	＜0.001

Index definition: **E-cigarettes are in 'Three No' states** China's e-cigarettes are in the state of "three noes". It means that there is no product standard, no quality supervision, and no safety evaluation.

© 2022 Huang P. et al.
